# Bayesian strategies: probabilistic programs as generalised graphical models

**DOI:** 10.1007/978-3-030-72019-3_19

**Published:** 2021-03-23

**Authors:** Hugo Paquet

**Affiliations:** Imperial College, London, UK; Department of Computer Science, University of Oxford, Oxford, UK

## Abstract

We introduce *Bayesian strategies*, a new interpretation of probabilistic programs in game semantics. This interpretation can be seen as a refinement of Bayesian networks.

Bayesian strategies are based on a new form of *event structure*, with two causal dependency relations respectively modelling control flow and data flow. This gives a graphical representation for probabilistic programs which resembles the concrete representations used in modern implementations of probabilistic programming.

From a theoretical viewpoint, Bayesian strategies provide a rich setting for denotational semantics. To demonstrate this we give a model for a general higher-order programming language with recursion, conditional statements, and primitives for sampling from continuous distributions and trace re-weighting. This is significant because Bayesian networks do not easily support higher-order functions or conditionals.

## 1 Introduction

One promise of probabilistic programming languages (PPLs) is to make Bayesian statistics accessible to anyone with a programming background. In a PPL, the programmer can express complex statistical models clearly and precisely, and they additionally gain access to the set of inference tools provided by the probabilistic programming system, which they can use for simulation, data analysis, *etc.* Such tools are usually designed so that the user does not require any in-depth knowledge of Bayesian inference algorithms.

A challenge for language designers is to provide efficient inference algorithms. This can be intricate, because programs can be arbitrarily complex, and inference requires a close interaction between the inference engine and the language interpreter [42, Ch.6]. In practice, many modern inference engines do not manipulate the program syntax direcly but instead exploit some *representation* of it, more suited to the type of inference method at hand (Metropolis-Hastings (MH), Sequential Monte Carlo (SMC), Hamiltonian Monte Carlo, variational inference, *etc.*).

While many authors have recently given proofs of correctness for inference algorithms (see for example [11,24,32]), most have focused on idealised descriptions of the algorithms, based on syntax or operational semantics, rather than on the concrete program representations used in practice. In this paper we instead put forward a mathematical semantics for probabilistic programs designed to provide reasoning tools for existing implementations of inference.

Our work targets a specific class of representations which we call *data flow* representations. We understand **data flow** as describing the dependence relationships between random variables of a program. This is in contrast with **control flow**, which describes *in what order* samples are performed. Such data flow representations are widely used in practice. We give a few examples. For Metropolis-Hastings inference, Church [30] and Venture [41] manipulate dependency graphs for random variables ("computation traces" or "probabilistic execution traces"); Infer.NET [22] compiles programs to *factor graphs* in order to apply message passing algorithms; for a subset of well-behaved programs, Gen [23] statically constructs a representation based on certain *combinators* which is then exploited by a number of inference algorithms; and finally, for variational inference, Pyro [9] and Edward [55] rely on data flow graphs for efficient computation of gradients by automatic differentiation. (Also [52,28].)

In this paper, we make a step towards correctness of these implementations and introduce **Bayesian strategies**, a new representation based on Winskel's event structures [46] which tracks *both* data flow and control flow. The Bayesian strategy corresponding to a program is obtained compositionally as is standard in concurrent game semantics [63], and provides an intensional foundation for probabilistic programs, complementary to existing approaches [24,57].

This paper was inspired by the pioneering work of Ścibior et al. [53], which provides the first denotational analysis for concrete inference representations. In particular, their work provides a general framework for proving correct inference algorithms based on static representations. But the authors do not show how their framework can be used to accommodate data flow representations or verify any of the concrete implementations mentioned above. The work of this paper does *not* fill this gap, as we make no attempt to connect our semantic constructions with those of [53], or indeed to prove correct any inference algorithms. This could be difficult, because our presentation arises out of previous work on game semantics and thus does not immediately fit in with the monadic techniques employed in [53]. Nonetheless, efforts to construct game semantics monadically are underway [14], and it is hoped that the results presented here will set the ground for the development of event structure-based validation of inference.

### 1.1 From Bayesian networks to Bayesian strategies

Consider the following basic model, found in the Pyro tutorials (and also used in [39]), used to infer the weight of an object based on two noisy measurements. The measurements are represented by random variables $meas_1$ and $meas_2$, whose values are drawn from a normal distribution around the true weight (*weight*), whose *prior* distribution is also normal, and centered at 2. (In this situation, $meas_1$ and $meas_2$ are destined to be *conditioned* on actual observed values, and the problem is then to infer the *posterior* distribution of *weight* based on these observations. We leave out conditioning in this example and focus on the model specification.)

To describe this model it is convenient to use a *Bayesian network*, *i.e.* a DAG of random variables in which the distribution of each variable depends only on the value of its parents:

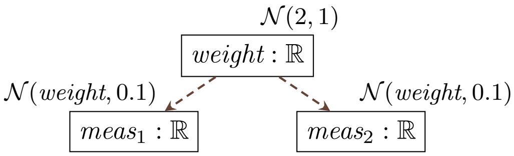

The same probabilistic model can be encoded in an ML-style language:

$$\begin{array}{l}
\textbf{let}\ \text{weight} = \textbf{sample}_{weight}\ \textbf{normal}(2,1)\ \textbf{in} \\
\textbf{sample}_{meas_1}\ \textbf{normal}(\text{weight}, 0.1); \\
\textbf{sample}_{meas_2}\ \textbf{normal}(\text{weight}, 0.1); \\
()
\end{array}$$

Our choice of sampling $meas_1$ before $meas_2$ is arbitrary: the same program with the second and third lines swapped corresponds to the same probabilistic model. This redundancy is unavoidable because programs are inherently sequential. It is the purpose of "commutative" semantics for probabilistic programs, as introduced by Staton et al. [54,57], to clarify this situation. They show that reordering program lines does not change the semantics, even in the presence of conditioning. This result says that when specifying a probabilistic model, only data flow matters, and not control flow. This motivates the use of program representations based on data flow such as the examples listed above.

In our game semantics, a probabilistic program is interpreted as a control flow graph *annotated* by a data dependency relation. The Bayesian strategy associated with the program above is as follows:

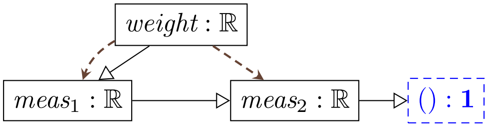

where (in brief), ⇢ is data flow, ⊸ is control flow, and the dashed node is the program output. (Probability distributions are as in the Bayesian network.)

The semantics is not commutative, simply because reordering lines affects control flow; we emphasise that the point of this work is not to prove any new program equations, but instead to provide a formal framework for the representations involved in practical inference settings.

### 1.2 Our approach

To formalise this idea we use event structures, which naturally model control flow, enriched with additional structure for probability and an explicit data flow relation. Event structures were used in previous work by the author and Castellan on probabilistic programming [18], and were shown to be a good fit for reasoning about MH inference. But the representation in [18] combines data flow and control flow in a single transitive relation, and thus suffers from important limitations. The present paper is a significant improvement: by maintaining a clear separation between control flow and data flow, we can reframe the ideas in the well-established area of *concurrent game semantics* [63], which enables an interpretation of recursion and higher-order functions; these were not considered in [18]. Additionally, here we account for the fact that data flow in probabilistic programming is *not* in general a transitive relation.

While there is some work in setting up the right notion of event structure, the standard methods of concurrent game semantics adapt well to this setting. This is not surprising, as event structures and games are known to be resistant to the addition of extra structure, see e.g. [21,5,15]. One difficulty is to correctly define composition, keeping track of potential hidden data dependencies. In summary:

– We introduce a general notion of Bayesian event structure, modelling control flow, data flow, and probability.
– We set up a compositional framework for these event structures based on concurrent games. Specifically, we define a category **BG** of *arenas* and *Bayesian strategies*, and give a description of its abstract properties.
– We give a denotational semantics for a higher-order statistical language. Our semantics gives an operationally intuitive representation for programs and their data flow structure, while only relying on standard mathematical tools.

*Paper outline.* We start by recalling the basics of probability and Bayesian networks, and we then describe the syntax of our language (Sec. 2). In Sec. 3, we introduce event structures and Bayesian event structures, and informally describe our semantics using examples. In Sec. 4 we define our category of arenas and strategies, which we apply to the denotational semantics of the language in Sec. 5. We give some context and perspectives in Sec. 6.

*Acknowledgements.* I am grateful to Simon Castellan, Mathieu Huot and Philip Saville for helpful comments on early versions of this paper. This work was supported by grants from EPSRC and the Royal Society.

## 2 Probability distributions, Bayesian networks, and probabilistic programming

### 2.1 Probability and measure

We recall the basic notions, see *e.g.* [8] for a reference.

*Measures.* A **measurable space** is a set $X$ equipped with a **$\sigma$-algebra**, that is, a set $\Sigma_X$ of subsets of $X$ containing $X$ itself, and closed under completements and countable unions. The elements of $\Sigma_X$ are called **measurable subsets** of $X$. An important example of measurable space is the set $\mathbb{R}$ equipped with its

$\sigma$-algebra $\Sigma_{\mathbb{R}}$ of Borel sets, the smallest one containing all intervals. Another basic example is the discrete space $\mathbb{N}$, in which all subsets are measurable.

A **measure** on $(X, \Sigma_X)$ is a function $\mu : \Sigma_X \to [0, \infty]$ which is countably additive, *i.e.* $\mu(\biguplus_{i\in I} U_i) = \sum_{i\in I} U_i$ for $I$ countable, and satisfies $\mu(\emptyset) = 0$. A fundamental example is the **Lebesgue measure** $\lambda$ on $\mathbb{R}$, defined on intervals as $\lambda([a, b]) = b - a$ and extended to all Borel sets. Another example (for arbitrary $X$) is the **Dirac measure** at a point $x \in X$: for any $U \in \Sigma_X$, $\delta_x(U) = 1$ if $x \in U$, 0 otherwise. A **sub-probability measure** on $(X, \Sigma_X)$ is a measure $\mu$ satisfying $\mu(X) \le 1$.

A function $f : X \to Y$ is measurable if $U \in \Sigma_Y \implies f^{-1}U \in \Sigma_X$. Given a measure on a space $X$ and a measurable function $f : X \to \mathbb{R}$, for every measurable subset $U$ of $X$ we can define the **integral** $\int_U \mathrm{d}\mu f$, an element of $\mathbb{R}\cup\{\infty\}$. This construction yields a measure on $X$. (Many well-known probability distributions on the reals arise in this way from their *density*.)

*Kernels.* We will make extensive use of *kernels*, which can be seen as parametrised families of measures. Formally a **kernel** from $X$ to $Y$ is a map $k : X \times \Sigma_Y \to [0, \infty]$ such that for every $x \in X$, $k(x, -)$ is a measure on $Y$, and for every $V \in \Sigma_Y$, $k(-, V)$ is a measurable function. It is a **sub-probability kernel** if each $k(x, -)$ is a sub-probability measure, and it is an **s-finite kernel** if it is a countable (pointwise) sum of sub-probability kernels. Every measurable function $f : X \to Y$ induces a Dirac kernel $\delta_f : X \rightsquigarrow Y : x \mapsto \delta_{f(x)}$. Kernels compose: if $k : X \rightsquigarrow Y$ and $h : Y \rightsquigarrow Z$ then the map $h \circ k : X \times \Sigma_Z \to [0, 1]$ defined as $(x, W) \mapsto \int_Y \mathrm{d}k(x, -)h(-, W)$ is also a kernel, and the Dirac kernel $\delta_{\mathrm{id}}$ (often just $\delta$) is an identity for this composition. We note that if both $h$ and $k$ are sub-probability kernels, then $h \circ k$ is a sub-probability kernel. Finally, observe that a kernel $\mathbf{1} \rightsquigarrow X$, for $\mathbf{1}$ a singleton space, is the same thing as a measure on $X$.

In this paper we will refer to the bernoulli, normal, and uniform families of distributions; all of these are sub-probability kernels from their parameters spaces to $\mathbb{N}$ or $\mathbb{R}$. For example, there is a kernel $\mathbb{R}^2 \rightsquigarrow \mathbb{R} : ((x, y), U) \mapsto \mu_{\mathcal{N}(x,y)}(U)$, where $\mu_{\mathcal{N}(x,y)}$ is the measure associated with a normal distribution with parameters $(x, y)$, if $y > 0$, and the 0 measure otherwise. We understand the bernoulli distribution as returning either 0 or $1 \in \mathbb{N}$.

*Product spaces and independence.* When several random quantities are under study one uses the notion of **product space**: given $(X, \Sigma_X)$ and $(Y, \Sigma_Y)$ we can equip the set $X \times Y$ with the product $\sigma$-algebra, written $\Sigma_{X\times Y}$, defined as the smallest one containing $U \times V$, for $U \in \Sigma_X$ and $V \in \Sigma_Y$.

A measure $\mu$ on $X \times Y$ gives rise to **marginals** $\mu_X$ and $\mu_Y$, measures on $X$ and $Y$ respectively, defined by $\mu_X(U) = \mu(U \times Y)$ and $\mu_Y(V) = \mu(X \times V)$ for $U \in \Sigma_X$ and $V \in \Sigma_Y$.

Given kernels $k : X \rightsquigarrow Y$ and $h : Z \rightsquigarrow W$ we define the **product kernel** $k \times h : X \times Z \rightsquigarrow Y \times W$ via iterated integration:

$$((x, z), U) \mapsto \int_{y\in Y} \mathrm{d}k(x, -) \int_{w\in W} \mathrm{d}h(z, -)\chi_U(y, w),$$

where $\chi_U$ is the characteristic function of $U \in \Sigma_{Y \times V}$. When $X = Z = \mathbf{1}$ this gives the notion of **product measure**.

The definitions above extend with no difficulty to product spaces $\prod_{i \in I} X_i$. A measure $P$ on $\prod_{i \in I} X_i$ has marginals $P_J$ for any $J \subseteq I$, and we say that $X_i$ and $X_j$ are **independent w.r.t. P** if the marginal $P_{i,j}$ is equal to the product measure $P_i \times P_j$.

### 2.2 Bayesian networks

An efficient way to define measures on product spaces is using probabilistic graphical models [37], for example Bayesian networks, whose definition we briefly recall now. The idea is to use a graph structure to encode a set of independence constraints between the components of a product space. We recall the definition of conditional independence. With respect to a joint distribution $P$ on $\prod_{i \in I} X_i$, we say $X_i$ and $X_j$ are **conditionally independent given** $X_k$ if there exists a kernel $k : X_k \rightsquigarrow X_i \times X_j$ such that $P_{i,j,k}(U_i \times U_j \times U_k) = \int_{U_k} k(-, U_i \times U_j) \mathrm{d}P_k$ for all measurable $U_i, U_j, U_k$, and $X_i$ and $X_j$ are independent w.r.t. $k(x_k, -)$ for all $x_k \in X_k$. In this definition, $k$ is a *conditional distribution* of $X_i \times X_j$ given $X_k$ (w.r.t. $P$); under some reasonable conditions [8] this always exists, and the independence condition is the main requirement.

Adapting the presentation used in [27], we define a **Bayesian network** as a directed acyclic graph $G = (V, \dashrightarrow)$ where each node $v \in V$ is assigned a measurable space $\mathcal{M}(v)$. We define the **parents** $\mathrm{pa}(v)$ of $v$ to be the set of nodes $u$ with $u \dashrightarrow v$, and its **non-descendants** $\mathrm{nd}(v)$ to contain the nodes $u$ such that there is no path $v \dashrightarrow \cdots \dashrightarrow u$. Writing $\mathcal{M}(S) = \prod_{v \in S} \mathcal{M}(v)$ for any subset $S \subseteq V$, a measure $P$ on $\mathcal{M}(V)$ is said to be **compatible with** $G$ if for every $v \in V$, $\mathcal{M}(v)$ and $\mathcal{M}(\mathrm{nd}(v))$ are independent given $\mathcal{M}(\mathrm{pa}(v))$. It is straightforward to verify that given a Bayesian network $G$, we can construct a compatible measure by supplying for every $v \in V$, an s-finite kernel $k_v : \mathcal{M}(\mathrm{pa}(v)) \rightsquigarrow \mathcal{M}(v)$.

(In practice, Bayesian networks are used to represent probabilistic models, and so typically every kernel $k_v$ is strictly probabilistic. Here the $k_v$ are only required to be s-finite, so they are in general unnormalised. As we will see, this is because we consider possibly *conditioned* models.)

Bayesian networks are an elegant way of constructing models, but they are limited. We now present a programming language whose expressivity goes beyond them.

### 2.3 A language for probabilistic modelling

Our language of study is a call-by-value statistical language with sums, products, and higher-order types, as well as recursive functions. Languages with comparable features are considered in [11,57,40].

The syntax of this language is described in Fig. 1. Note the distinction between general terms $M, N$ and values $V$. The language includes the usual term constructors and pattern matching. Base types are the unit type, the real numbers and the natural numbers, and for each of them there are associated constants. The language is parametrised by a set $\mathcal{L}$ of *labels*, a set $\mathcal{F}$ of partial measurable functions $\mathbb{R}^n \to \mathbb{R}$ or $\mathbb{R}^n \to \mathbb{N}$, and a set $\mathcal{D}$ of standard distribution families, which are sub-probability kernels[1] $\mathbb{R}^n \rightsquigarrow \mathbb{R}$ or $\mathbb{R}^n \rightsquigarrow \mathbb{N}$. There is also a primitive **score** which multiplies the weight of the current trace by the value of its argument. This is an idealised form of conditioning via *soft constraints*, which justifies the move from sub-probability to s-finite kernels (see [54]).

$$
\begin{aligned}
A, B &::= \mathbf{1} \mid \mathbb{N} \mid \mathbb{R} \mid A \times B \mid A + B \mid A \to B \\
V, W &::= () \mid \underline{n} \mid \underline{r} \mid \underline{f} \mid (V, W) \mid \mathbf{inl}\, V \mid \mathbf{inr}\, V \mid \lambda x.M \\
M, N &::= V \mid x \mid M\, N \mid M =^{?} 0 \mid \mu x : A \to B.M \mid \mathbf{sample}_{\ell}\ \mathbf{dist}(M_1, \ldots, M_n) \\
&\quad\ (M, N) \mid \mathbf{match}\ M\ \mathbf{with}\ (x, y) \to P \mid \mathbf{score}\ M \\
&\quad\ \mathbf{inl}\, M \mid \mathbf{inr}\, M \mid \mathbf{match}\ M\ \mathbf{with}\ [\mathbf{inl}\, x \to N_1 \mid \mathbf{inr}\, x \to N_2]
\end{aligned}
$$

Fig. 1: Syntax.

$$
\frac{r \in \mathbb{R}}{\Gamma \vdash \underline{r} : \mathbb{R}} \qquad
\frac{\Gamma \vdash M : \mathbb{N}}{\Gamma \vdash M =^{?} 0 : \mathbb{B}} \qquad
\frac{\Gamma \vdash M : \mathbb{R}}{\Gamma \vdash \mathbf{score}\ M : \mathbf{1}} \qquad
\frac{\Gamma, x : A \to B \vdash M : A \to B}{\Gamma \vdash \mu x : A \to B.M : A \to B}
$$

$$
\frac{(f : \mathbb{R}^n \rightharpoonup \mathbb{X}) \in \mathcal{F}}{\Gamma \vdash \underline{f} : \mathbb{R}^n \to \mathbb{X}} \qquad
\frac{(\mathbf{dist} : \mathbb{R}^n \to \mathbb{X}) \in \mathcal{D} \quad \text{For } i = 1, \ldots, n,\ \Gamma \vdash M_i : \mathbb{R} \quad \ell \in \mathcal{L}}{\Gamma \vdash \mathbf{sample}_{\ell}\ \mathbf{dist}(M_1, \ldots, M_n) : \mathbb{X}}
$$

Fig. 2: Subset of typing rules.

Terms of the language are typed in the standard way; in Fig. 2 we present a subset of the rules which could be considered non-standard. We use $\mathbb{X}$ to stand for either $\mathbb{N}$ or $\mathbb{R}$, and we do not distinguish between the type and the corresponding measurable space. We also write $\mathbb{B}$ for $\mathbf{1} + \mathbf{1}$, and use syntactic sugar for let-bindings, sequencing, and conditionals:

$$
\begin{aligned}
\mathbf{let}\ x : A = M\ \mathbf{in}\ N \quad &:= \quad (\lambda x : A.N)\, M \\
M; N \quad &:= \quad \mathbf{let}\ x : A = M\ \mathbf{in}\ N \quad (\text{for } x \text{ not free in } N) \\
\mathbf{if}\, M\ \mathbf{then}\ N_1\ \mathbf{else}\ N_2 \quad &:= \quad \mathbf{match}\ M\ \mathbf{with}\ [\mathbf{inl}\, x \to N_1 \mid \mathbf{inr}\, x \to N_2]
\end{aligned}
$$

## 3 Programs as event structures

In this section, we introduce our causal approach. We give a series of examples illustrating how programs can be understood as graph-like structures known as *event structures*, of which we assume no prior knowledge. Event structures were introduced by Winskel et al. [46], though for the purposes of this work the traditional notion must be significantly enriched.

[1] In any practical instance of the language it would be expected that every kernel in $\mathcal{D}$ has a density in $\mathcal{F}$, but this is not strictly necessary here.

$\textbf{let}\ \text{weight} = \textbf{sample}_{weight}\ \textbf{normal}(2, 1)\ \textbf{in}$
$\textbf{sample}_{meas_1}\ \textbf{normal}(\text{weight}, 0.1);$
$\textbf{sample}_{meas_2}\ \textbf{normal}(\text{weight}, 0.1); ()$

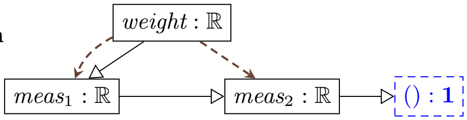

Fig. 3

The examples which follow are designed to showcase the following features of the semantics: combination of data flow and control flow with probability (Sec. 3.1), conditional branching (Sec. 3.2), open programs with multiple arguments (Sec. 3.3) and finally higher-order programs (Sec. 3.4). We will then give further definitions in Sec. 3.5 and Sec. 3.6.

Our presentation in Sec. 3.1 and Sec. 3.2 is intended to be informal; we give all the necessary definitions starting from Sec. 3.3.

### 3.1 Control flow, data flow, and probability

We briefly recall the example of the introduction; the program and its semantics are given in Fig. 3. As before, $\rightarrowtriangle$ represents control flow, and $\dashrightarrow$ represents data flow. There is a node for each random choice in the program, and the dependency relationships are pictured using the appropriate arrows. Naturally, a data dependency imposes constraints on the control flow: every arrow $\dashrightarrow$ must be realised by a control flow path $\rightarrowtriangle^*$. There is an additional node for the output value, drawn in a dashed box, which indicates that it is a possible point of interaction with other programs. This will be discussed in Sec. 3.3.

Although this is not pictured in the above diagram, the semantics also comprises a family of kernels, modelling the probabilistic execution according to the distributions specified by the program. Intuitively, each node has a distribution whose parameters are its parents for the relation $\dashrightarrow$. For example, the node labelled $meas_2$ will be assigned a kernel $k_{meas_2} : \mathbb{R} \rightsquigarrow \mathbb{R}$ defined so that $k_{meas_2}(weight, -)$ is a normal distribution with parameters $(weight, 0.1)$.

### 3.2 Branching

Consider a modified scenario in which only one measurement is performed, but with probability 0.01 an error occurs and the scales display a random number between 0 and 10. The corresponding program and its semantics are given in Fig. 4.

In order to represent the conditional statement we have introduced a new element to the graph: a binary relation known as *conflict*, pictured $\sim\!\sim\!\sim$, and indicating that two nodes are incompatible and any execution of the program will only encounter one of them. Conflict is *hereditary*, in the sense that the respective futures of two nodes in conflict are also incompatible. Hence we need two copies of $()$; one for each branch of the conditional statement. Unsurprisingly,

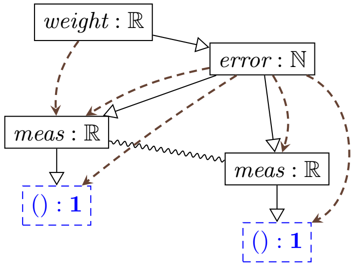

**let** weight = **sample**$_{weight}$ **normal**(2, 1) **in**
**let** error = **sample**$_{error}$ **bernoulli**(0.01) **in**
**if** error $=^?$ 0
 **then sample**$_{meas}$ **uniform**(0, 10)
 **else sample**$_{meas}$ **normal**(weight, 0.1); ()

Fig. 4

beyond the branching point all events depend on *error*, since their very existence depends on its value.

We continue our informal presentation with a description of the semantics of open terms. This will provide enough context to formally define the notion of event structure we use in this paper, which differs from others found in the literature.

### 3.3 Programs with free variables

We turn the example in Sec. 3.2 into one involving two free variables, *guess* and *rate*, used as parameters for the distributions of *weight* and *error*, respectively. These allow the same program to serve as a model for different situations. Formally we have a term $M$ such that $guess : \mathbb{R}, rate : \mathbb{R} \vdash M : \mathbf{1}$, given in Fig. 5 with its semantics. We see that the two parameters are themselves represented

**let** weight = **sample**$_{weight}$ **normal**(*guess*, 1) **in**
**let** error = **sample**$_{error}$ **bernoulli**(*rate*) **in**
**if** error $=^?$ 0
 **then sample**$_{meas}$ **uniform**(0, 10)
 **else sample**$_{meas}$ **normal**(weight, 0.1); ()

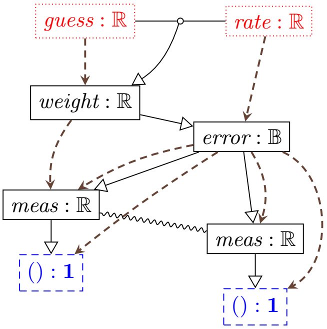

Fig. 5

by nodes, drawn in dotted boxes, showing that (like the output nodes) they are a point of interaction with the program's external environment; this time, a value is received rather than sent. Below, we will distinguish between the different types of nodes by means of a *polarity* function.

We attach to the parameter nodes the appropriate data dependency arrows. The subtlety here is with control flow: while is it clear that parameter values must be obtained before the start of the execution, and that necessarily $guess \rightarrow weight$ and $rate \rightarrow weight$, it is less clear what relationship *guess* and *rate* should have with each other.

In a call-by-value language, we find that leaving program arguments causally independent (of each other) leads to soundness issues. But it would be equally unsound to impose a causal order between them. Therefore, we introduce a form of synchronisation relation, amounting to having both $\mathit{guess} \rightarrowtriangle \mathit{rate}$ and $\mathit{rate} \rightarrowtriangle \mathit{guess}$, but we write $\mathit{guess} \mathrel{-\!\!\circ\!\!-} \mathit{rate}$ instead. In event structure terminology this is known as a *coincidence*, and was introduced by [19] to study the synchronous $\pi$-calculus. Note that in many approaches to call-by-value games (*e.g.* [31,26]) one would bundle both parameters into a single node representing the pair $(\mathit{guess}, \mathit{rate})$, but this is not suitable here since our data flow analysis requires separate nodes.

We proceed to define event structures, combining the ingredients we have described so far: control dependency, data dependency, conflict, and coincidence, together with a *polarity* function, used implicitly above to distinguish between input nodes ($-$), output nodes ($+$), and internal random choices ($0$).

**Definition 1.** *An* ***event structure*** $E$ *is a set* $E$ *of* events *(or* nodes*) together with the following structure:*

- *A* ***control flow preorder*** $\leq$ *on* $E$*, and such that each event has a finite history:* $\forall e \in E$*, the set* $[e] := \{e' \in E \mid e' \leq e\}$ *is finite. This preorder is designed to be generated from the* ***immediate dependency relation*** $\rightarrowtriangle$ *and the* ***coincidence relation*** $\mathrel{-\!\!\circ\!\!-}$*, which can both be recovered from* $\leq$*, as follows: we write* $e \mathrel{-\!\!\circ\!\!-} e'$ *when* $e$ *and* $e'$ *are equivalent in the preorder,* i.e. $e \leq e'$ *and* $e' \leq e$*; and* $e \rightarrowtriangle e'$ *whenever the following holds:* $e < e'$*,* $\neg(e' > e)$*, and if* $e \leq d \leq e'$ *then either* $d \mathrel{-\!\!\circ\!\!-} e$ *or* $d \mathrel{-\!\!\circ\!\!-} e'$*; .*
- *An irreflexive, binary* ***conflict relation*** $\#$ *on* $E$*, which is* hereditary*: if* $e \leq e'$ *and* $e \# d$ *then* $e' \# d$*. Observe that this applies when* $e \mathrel{-\!\!\circ\!\!-} e'$*. The* ***minimal conflict relation*** $\sim\!\sim$ *(typically used in diagrams) is defined as follows:* $e \sim\!\sim d$ *if* $e \# d$*, but for every* $d_0 < d$ *and* $e_0 < e$*,* $\neg(e \# d_0)$ *and* $\neg(e_0 \# d)$*.*
- *An irreflexive, binary* ***data flow relation*** $\dashrightarrow$ *on* $E$*, such that if* $e \dashrightarrow e'$ *then* $e \leq e'$ *and* $\neg(e \mathrel{-\!\!\circ\!\!-} e')$*. Note that this is not required to be transitive.*
- *A* ***polarity function*** $\mathit{pol} : E \to \{+, 0, -\}$*, such that if* $e \mathrel{-\!\!\circ\!\!-} e'$ *then* $\mathit{pol}(e) = \mathit{pol}(e') \neq 0$*.*
- *A* ***labelling function*** $\mathrm{lbl} : E_0 \to \mathcal{L}$*, defined on the set* $E_0 := \{e \in E \mid \mathit{pol}(e) = 0\}$*.*

Often we write $E$ instead of the whole tuple $(E, \leq, \#, \dashrightarrow, \mathrm{pol})$. It is sometimes useful to quotient out coincidences: we write $E_{\mathrel{-\!\circ\!-}}$ for the **set of $\mathrel{-\!\!\circ\!\!-}$-equivalence classes**, which we denote as boldface letters $(\mathbf{e}, \mathbf{a}, \mathbf{s}, \ldots)$. It is easy to check that this is also an event structure with $\mathbf{e} \leq \mathbf{e}'$ (resp. $\#, \dashrightarrow$) if there is $e \in \mathbf{e}$ and $e' \in \mathbf{e}'$ with $e \leq e'$ (resp. $\#, \dashrightarrow$), and evident polarity function.

We will see in Sec. 3.5 how this structure can be equipped with quantitative information (in the form of measurable spaces and kernels). Before discussing higher-order programs, we introduce the fundamental concept of *configuration*, which will play an essential role in the technical development of this paper.

**Definition 2.** *A **configuration** of $E$ is a finite subset $x \subseteq E$ which is down-closed (if $e \leq e'$ and $e' \in x$ then $e \in x$) and conflict-free (if $e, e' \in x$ then $\neg(e \# e')$). The **set of all configurations** of $E$ is denoted $\mathscr{C}(E)$ and it is a partial order under $\subseteq$.*

We introduce some important terminology. For an event $e \in E$, we have defined its **history** $[e]$ above. This is always a configuration of $E$, and the smallest one containing $e$. More generally we can define $[\mathbf{e}] = \{e' \mid \forall e \in \mathbf{e}.\, e' \leq e\}$, and $[\mathbf{e}) = [\mathbf{e}] \setminus \mathbf{e}$.

The **covering relation** $-\!\!\subset$ defines the smallest non-trivial extensions to a configuration; it is defined as follows: $x -\!\!\subset y$ if there is $\mathbf{e} \in E_{\multimap}$ such that $x \cap \mathbf{e} = \emptyset$ and $y = x \cup \mathbf{e}$. We will sometimes write $x -\!\!\subset^{\mathbf{e}} y$. We sometimes annotate $-\!\!\subset$ and $\subseteq$ with the polarities of the added events: so for instance $x \subseteq_{+,0} y$ if each $e_i \in y \setminus x$ has polarity $+$ or $0$.

### 3.4 Higher-order programs

We return to a fairly informal presentation; our goal now is to convey intuition about the representation of higher-order programs in the framework of event structures. We will see in Sec. 4 how this representation is obtained from the usual categorical approach to denotational semantics.

Consider yet another faulty-scales scenario, in which the probability of error now depends on the object's weight. Suppose that this dependency is not known by the program, and thus left as a parameter $rate : \mathbb{R} \to \mathbb{R}$. The resulting program has type $rate : \mathbb{R} \to \mathbb{R}, guess : \mathbb{R} \vdash \mathbb{R}$, as follows:

$$\textbf{let}\ \mathrm{weight} = \textbf{sample}_{weight}\ \textbf{normal}(guess, 1)\ \textbf{in}$$
$$\textbf{let}\ \mathrm{error} = \textbf{sample}_{error}\ \textbf{bernoulli}\ (rate\ \mathrm{weight})\ \textbf{in}\ \mathrm{error}$$

We give its semantics in Fig. 6. (To keep things simple this scenario involves no measurements.)

It is an important feature of the semantics presented here that higher-order programs are interpreted as causal structures involving only values of ground type. In the example, the argument *rate* is initially received not as a mathematical function, but as a single message of unit type (labelled $\lambda^{rate}$), which gives the program the possibility to call the function *rate* by feeding it an input value. Because the behaviour of *rate* is unknown, its output is treated as a new argument to the program, represented by the negative *out* node. The shaded region

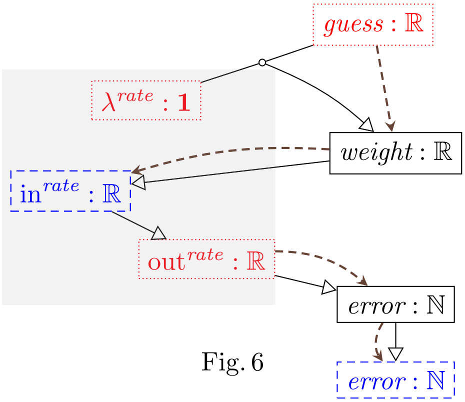

Fig. 6

highlights the part of computation during which the program interacts with its argument *rate*. The semantics accommodates the possibiliy that *rate* itself has internal random choices; this will be accounted for in the compositional framework of Sec. 4.

### 3.5 Bayesian event structures

We show now that event structures admit a probabilistic enrichment.[2]

**Definition 3.** *A* ***measurable event structure*** *is an event structure together with the assignment of a measurable space* $\mathcal{M}(e)$ *for every event* $e \in E$*. For any* $X \subseteq E$ *we set* $\mathcal{M}(X) = \prod_{e \in X} \mathcal{M}(e)$.

As is common in statistics, we often call $\underline{e}$ (or $\underline{X}$) an element of $\mathcal{M}(e)$ (or $\mathcal{M}(X)$). We now proceed to equip this with a kernel for each event.

**Definition 4.** *For* $E$ *an event structure and* $e \in E$*, we define the* ***parents*** $\mathrm{pa}(e)$ *of* $e$ *as* $\{d \in E \mid d \dashrightarrow e\}$.

**Definition 5.** *A* ***quantitative event structure*** *is a measurable event structure* $E$ *with, for every non-negative* $e \in E$*, a kernel* $k_e : \mathcal{M}(\mathrm{pa}(e)) \rightsquigarrow \mathcal{M}(e)$.

Our *Bayesian* event structures are quantitative event structures satisfying an additional axiom, which we introduce next. This axiom is necessary for a smooth combination of data flow and control flow; without it, the compositional framework of the next section is not possible.

**Definition 6.** *Let* $E$ *be a quantitative event structure. We say that* $e \in E$ *is* ***non-uniform*** *if there are distinct* $\underline{\mathrm{pa}}(e), \underline{\mathrm{pa}}'(e) \in \mathcal{M}(\mathrm{pa}(e))$ *such that*

$$k_e(\underline{\mathrm{pa}}(e), \mathcal{M}(e)) \neq k_e(\underline{\mathrm{pa}}'(e), \mathcal{M}(e)).$$

We finally define:

**Definition 7.** *A* ***Bayesian event structure*** *is a quantitative event structure such that if* $e \in E$ *is non-uniform, and* $e \leq e'$ *with* $e$ *and* $e'$ *not coincident, then* $\mathrm{pa}(e) \subseteq \mathrm{pa}(e')$.

The purpose of this condition is to ensure that Bayesian event structures support a well-behaved notion of "hiding", which we will define in the next section.

### 3.6 Symmetry

For higher-order programs, event structures in the sense of Definition 1 present a limitation. This has to do with the possibility for a program to call a function argument more than once, which the compositional framework of Sec. 4 does not readily support. We will use a linear logic-inspired "!" to duplicate nodes, thus making certain configurations available in infinitely many copies. The following additional structure, called *symmetry*, is there to enforce that these configurations yield equivalent behaviour.

[2] We emphasise that our notion of "event" is *not* related to the usual notion of event in probability theory.

**Definition 8 (Winskel [61]).** *A* ***symmetry*** *on an event structure $E$ is a family $\cong_E$ of bijections $\theta : x \cong y$, with $x, y \in \mathscr{C}(E)$, containing all identity bijections and closed under composition and inverses, satisfying the following axioms.*

- *For each $\theta : x \cong y$ in $\cong_E$, if $x \subseteq x'$ then there is a bijection $\theta' : x' \cong y'$ in $\cong_E$, such that $\theta \subseteq \theta'$. The analogous property is required for every restriction $x' \subseteq x$.*
- *Each $\theta \in \cong_E$ preserves polarity ($pol(e) = pol(\theta(e))$), data flow ($e \dashrightarrow e' \implies \theta(e) \dashrightarrow \theta(e')$), and measurable structure ($\mathcal{M}(e) = \mathcal{M}(\theta(e))$).*

*We write $\theta : x \cong_E y$ if $(\theta : x \cong y) \in \cong_E$. When $E$ is Bayesian, we additionally require $k_e = k_{\theta(e)}$ for every non-negative $e \in x$. (This is well-defined because $\theta$ preserves data flow and thus* $\mathrm{pa}(\theta(\mathbf{e})) = \theta\,\mathrm{pa}(\mathbf{e})$*.)*

Although symmetry can be mathematically subtle, combining it with additional data on event structures does not usually pose any difficulty [15,48].

In this section we have described Bayesian event structures with symmetry, which are the basic mathematical objects we use to represent programs. A central contribution of this paper is to define a *compositional* semantics, in which the interpretation of a program is obtained from that of its sub-programs. This is the topic of the next section.

## 4 Games and Bayesian strategies

The presentation is based on *game semantics*, a line of research in the semantics of programming languages initiated in [3,33], though the subject has earlier roots in the semantics of linear logic proofs (e.g. [10]).

It is typical of game semantics that programs are interpreted as concrete computational trees, and that higher-order terms are described in terms of the possible interactions with their arguments. As we have seen in the examples of the previous section, this interaction takes the form of an exchange of first-order values. The central technical achievement of game semantics is to provide a method for *composing* such representations.

To the reader not familiar with game semantics, the terminology may be misleading: the work of this paper hardly retains any connection to game theory. In particular there is no notion of *winning*. The analogy may be understood as follows for a given program of type $\Gamma \vdash M : A$. There are two players: the program itself, and its environment. The "game", which we study from the point of view of the program, takes place in the *arena* $[\![\Gamma \vdash A]\!]$, which specifies which moves are allowed (calls to arguments in $\Gamma$, internal samples, return values in $A$, *etc.*). The semantics of $M$ is a *strategy* (written $[\![M]\!]$), which specifies a plan of action for the program to follow in reaction to the moves played by the environment; this plan has to obey the constraints specified by the arena.

### 4.1 An introduction to game semantics based on event structures

There are many formulations of game semantics in the literature, with varying advantages. This paper proposes to use *concurrent games*, based on event structures, for reasoning about data flow in probabilistic programs. Originally introduced in [51] (though some important ideas appeared earlier: [25,44]), concurrent games based on event structures have been extensively developed and have found a range of applications.

In Sec. 2, we motivated our approach by assigning event structures to programs; these event structures are examples of *strategies*, which we will shortly define. First we define *arenas*, which are the objects of the category we will eventually build. (The morphisms will be strategies.)

Perhaps surprisingly, an arena is also defined as an event structure, though a much simpler one, with no probabilistic information, empty data dependency relation $\dashrightarrow$, and no neutral polarity events. We call this a **simple event structure**. This event structure does not itself represent any computation, but is simply there to constrain the shape of strategies, just as types constrain programs. Before giving the definition, we present in Fig. 7 the arenas associated with the strategies in Sec. 3.3 and Sec. 3.4, stating which types they represent. Note the **copy indices** (0, 1, ...) in Fig. 7b; these point to duplicated (*i.e. symmetric*) branches.

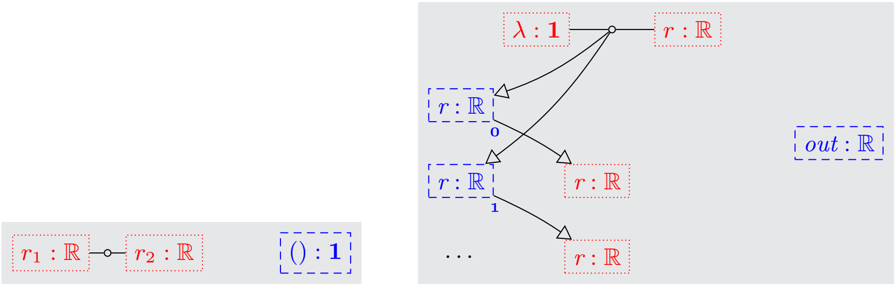

(a) The arena $[\![\mathbb{R}, \mathbb{R} \vdash \mathbf{1}]\!]$.

(b) The arena $[\![\mathbb{R} \to \mathbb{R}, \mathbb{R} \vdash \mathbb{R}]\!]$.

Fig. 7: Examples of arenas.

**Definition 9.** *An* ***arena*** *is a simple, measurable event structure with symmetry* $\mathcal{A} = (A, \cong_A)$, *together with two sub-symmetries* $\cong_A^+$ *and* $\cong_A^-$, *subject to the following conditions:*

- *$A$ is a simple event structure which is* ***alternating****: if $a \rightarrowtriangle b$ then $pol(a) \neq pol(b)$;* ***forest-shaped****: if $a \leq b$ and $c \leq b$ then $a \leq c$ or $c \leq a$ (or both); and* ***race-free****: if $a \rightsquigarrow b$ then $pol(a) = pol(b)$.*
- *$\cong_A$, $\cong_A^-$ and $\cong_A^+$ satisfy the axioms of* thin concurrent games *[17, 3.17].*
- *If $a, a'$ are symmetric moves (*i.e. *there is $\theta \in \cong_A$ such that $\theta(a) = a'$) then $\mathcal{M}(a) = \mathcal{M}(a')$.*

*Write $init(A)$ for the set of* ***initial events****,* i.e. *those minimal for $\leq$. We say that $\mathcal{A}$ is* ***positive*** *if every $a \in init(A)$ is positive. (****Negative*** *arenas are defined*

*similarly.) We say that $\mathcal{A}$ is* ***regular*** *if whenever $a, b \in init(A)$, either $a \multimap b$ or $a \rightsquigarrow b$.*

So, arenas provide a set of moves together with certain constraints for playing those moves. Our definition of strategy is slightly technical, but the various conditions ensure that strategies can be composed soundly; we will explore this second point in Sec. 4.2.

For a strategy $S$ to be well-defined relative to an arena $A$, each positive or negative move of $S$ must correspond to a move of $A$; however neutral moves of $S$ correspond to internal samples of the program; these should not be constrained by the type. Accordingly, a strategy comprises a partial map $S \rightharpoonup A$ defined precisely on the non-neutral events. The reader should be able to reconstruct this map for the examples of Sec. 3.3 and Sec. 3.4.

**Definition 10.** *A* ***strategy*** *on an arena $\mathcal{A}$ is a Bayesian event structure with symmetry $\mathcal{S} = (S, \cong_S)$, together with a partial function $\sigma : S \rightharpoonup A$, whose domain of definition is exactly the subset $\{s \in S \mid pol(s) \neq 0\}$, and such that whenever $\sigma(s)$ is defined, $\mathcal{M}(\sigma(s)) = \mathcal{M}(s)$ and $pol(\sigma(s)) = pol(s)$. This data is subject to the following additional conditions:*

*(1) $\sigma$* ***preserves configurations****: if $x \in \mathscr{C}(S)$ then $\sigma x \in \mathscr{C}(A)$; and is* ***locally injective****: for $s, s' \in x \in \mathscr{C}(S)$, if $\sigma(s) = \sigma(s')$ then $s = s'$.*
*(2) $\sigma$ is* ***courteous****: if $s \rightarrowtriangle s'$ in $S$ and either $pol(s) = +$ or $pol(s') = -$, then $\sigma(s) \rightarrowtriangle \sigma(s')$.*
*(3) $\sigma$* ***preserves symmetry*** *($\theta : x \cong_S y \implies \sigma\theta : \sigma x \cong_A \sigma y$), and it is* ***$\cong$-receptive****: if $\theta : x \cong_S y$ and $\sigma\theta \multimap\!\!\!\subset_- \psi \in \cong_A$ then there exists a unique $\theta' \in \cong_S$ such that $\theta \multimap\!\!\!\subset_- \theta'$ and $\sigma\theta' = \psi$ ; and* ***thin****: if $x \in \mathscr{C}(S)$ and $\mathrm{id}_x \multimap\!\!\!\subset_{+,0} \theta$ for some $\theta \in \cong_S$, then $\theta = \mathrm{id}_{x'}$ for some $x' \in \mathscr{C}(S)$.*
*(4) If $s \dashrightarrow s'$ in $S$, then $pol(s') \neq -$ and $pol(s) \neq +$.*

Condition (1) amounts to $\sigma$ being a map of event structures [60]. Combined with (2) and (3), we get the usual notion of a concurrent strategy on an arena with symmetry [17]; and finally (4) is a form of $\dashrightarrow$-courtesy.

To these four conditions we add the following:

**Definition 11.** *A strategy $\mathcal{S}$ is* ***innocent*** *if conflict is local: $s \rightsquigarrow s' \implies [s) = [s')$, and for every $s \in S$, the following conditions hold:*

- *(*backwards sequentiality*) the history $[s]$ is a total preorder; and*
- *(*forward sequentiality*) if $[s] \multimap\!\!\!\subset^{\mathbf{s}_1}_{0,+}$ and $[s] \multimap\!\!\!\subset^{\mathbf{s}_2}_{0,+}$ and $\mathbf{s}_1 \neq \mathbf{s}_2$, then $\mathbf{s}_1 \rightsquigarrow \mathbf{s}_2$.*

Innocence [33,56,16] prevents any non-local or concurrent behaviour. It is typically used to characterise "purely functional" sequential programs, *i.e.* those using no state or control features. Here, we use innocence as a way to confine ourselves to a simpler semantic universe. In particular we avoid the need to deal with the difficulties of combining concurrency and probability [62].

In the rest of the paper, a **Bayesian strategy** is an innocent strategy in the sense of Definition 10 and Definition 11.

### 4.2 Composition of strategies

At this point, we have seen how to define *arenas*, and we have said that the event structures of Sec. 2 arise as *strategies* $\sigma : \mathcal{S} \rightharpoonup \mathcal{A}$ for an arena $\mathcal{A}$. As usual in denotational semantics, these will be obtained compositionally, by induction on the syntax. For this we must move to a categorical setting, in which arenas are objects and strategies are morphisms.

**Strategies as morphisms.** Before we introduce the notion of strategy *from $\mathcal{A}$ to $\mathcal{B}$* we must introduce some important construction on event structures.

**Definition 12.** *If $A$ is an event structure, its* ***dual*** *$A^\perp$ is the event structure whose structure is the same as $A$ but for polarity, which is defined at $pol_{A^\perp}(a) = -pol_A(a)$. (Negative moves become positive, and vice-versa, with neutral moves not affected.) For arenas, we define $(A, \cong_A, \cong_A^-, \cong_A^+)^\perp = (A^\perp, \cong_A, \cong_A^+, \cong_A^-)$.*

*Given a family $(\mathcal{A}_i)_{i\in I}$ of event structures with symmetry, we define their* ***parallel composition*** *to have events $\|_{i\in I} A_i = \bigcup_{i\in I} A_i \times \{i\}$ with polarity, conflict and both kinds of dependency obtained componentwise. Noticing that a configuration $x \in \mathscr{C}(\|_{i\in I} A_i)$ corresponds to $\|_{i\in I} x_i$ where each $x_i \in \mathscr{C}(A_i)$, and $x_i = \emptyset$ for all but finitely many $i$, we define the symmetry $\cong_{\|_{i\in I} A_i}$ to contain bijections $\|_i \theta_i : \|_i x_i \cong \|_i y_i$ where each $\theta_i \in \cong_{A_i}$. If the $\mathcal{A}_i$ are arenas we define the two other symmetries in the same way.*

We can now define our morphisms: a **strategy from $\mathcal{A}$ to $\mathcal{B}$** is a strategy on the arena $\mathcal{A}^\perp \parallel \mathcal{B}$, *i.e.* a map $\sigma : \mathcal{S} \rightharpoonup \mathcal{A}^\perp \parallel \mathcal{B}$. The event structure $S$ consists of $A$-*moves* (those mapped to the $A^\perp$ component), $B$-*moves*, and internal (*i.e.* neutral) events. We sometimes write $\mathcal{S} : A \nrightarrow B$.

The purpose of the composition operation $\odot$ which we proceed to define is therefore to produce, from a pair of strategies $\sigma : \mathcal{S} \rightharpoonup \mathcal{A}^\perp \parallel \mathcal{B}$ and $\tau : \mathcal{T} \rightharpoonup \mathcal{B}^\perp \parallel \mathcal{C}$, a strategy $\tau \odot \sigma : \mathcal{T} \odot \mathcal{S} \rightharpoonup \mathcal{A}^\perp \parallel \mathcal{C}$. A constant feature of denotational games models is that composition is defined in two steps: *interaction*, in which $\mathcal{S}$ and $\mathcal{T}$ synchronise by playing matching $B$-moves, and *hiding*, where the matching pairs of events are deleted. The setting of this paper allows both $\sigma$ and $\tau$ to be partial maps, so that in general there can be neutral events in both $\mathcal{S}$ and $\mathcal{T}$; these *never* synchronise, and indeed they should not be hidden, since we aim to give an account of internal sampling.

Before moving on to composition, a word of warning: the resulting structure will *not* be a category. Instead, arenas and strategies assemble into a weaker structure called a *bicategory* [6]. Bicategories have objects, morphisms, and *2-cells* (morphisms between morphisms), and the associativity and identity laws are relaxed, and only need to hold up to isomorphisms. (This situation is relatively common for intensional models of non-determinism.)

**Definition 13.** *Two strategies $\sigma : \mathcal{S} \rightharpoonup \mathcal{A}^\perp \parallel \mathcal{B}$ and $\sigma' : \mathcal{S}' \rightharpoonup \mathcal{A}^\perp \parallel \mathcal{B}$ are* ***isomorphic*** *if there is a bijection $f : S \cong S'$ preserving all structure, and such that for every $x \in \mathscr{C}(S)$, the bijection with graph $\{(\sigma(s), \sigma'(f(s))) \mid s \in x\}$ is in $\cong_A^+$.*

Intuitively, $\mathcal{S}$ and $\mathcal{S}'$ have the same moves up to the choice of copy indices. We know from [17] that isomorphism is preserved by composition (and all other constructions), so from now on we always consider strategies up to isomorphism; then we will get a category.

**Interaction.** In what follows we assume fixed Bayesian innocent strategies $\mathcal{S} : \mathcal{A} \nrightarrow \mathcal{B}$ and $\mathcal{T} : \mathcal{B} \nrightarrow \mathcal{C}$ as above, and study their interaction. We have hinted at the concept of "matching events" but the more convenient notion is that of *matching configurations*, which we define next.

**Definition 14.** *Configurations $x_S \in \mathscr{C}(S)$ and $x_T \in \mathscr{C}(T)$ are **matching** if there are $x_A \in \mathscr{C}(A)$ and $x_C \in \mathscr{C}(C)$ such that $\sigma x_S \parallel x_C = x_A \parallel \tau x_T$.*

There is an event structure with symmetry $\mathcal{T} \circledast \mathcal{S}$ whose configurations correspond precisely to matching pairs; it is a well-known fact in game semantics that innocent strategies compose "like relations" [43,15]. Because "matching" $B$-moves have a different polarity in $\mathcal{S}$ and $\mathcal{T}$, there is an ambiguity in the polarity of some events in $T \circledast S$; we address this after the lemma.

**Lemma 1.** *Ignoring polarity, there is, up to isomorphism, a unique event structure with symmetry $\mathcal{T} \circledast \mathcal{S}$, such that:*

- *There is an order-isomorphism $\mathscr{C}(T \circledast S) \cong \{(x_S, x_T) \in \mathscr{C}(S) \times \mathscr{C}(T) \mid x_S \text{ and } x_T \text{ matching}\}$. Write $x_T \circledast x_S$ for the configuration corresponding to $(x_S, x_T)$.*
- *There are partial functions $\Pi_S : T \circledast S \rightharpoonup S$ and $\Pi_T : T \circledast S \rightharpoonup T$, such that for every $x_T \circledast x_S \in \mathscr{C}(T \circledast S)$, $\Pi_S(x_T \circledast x_S) = x_S$ and $\Pi_S(x_T \circledast x_S) = x_T$.*
- *For every $e, e' \in T \circledast S$, $e \rightarrowtriangle e'$ iff either $\Pi_S(e) \rightarrowtriangle \Pi_S(e')$ or $\Pi_T(e) \rightarrowtriangle \Pi_T(e')$, and the same property holds for the conflict and data dependency relations.*
- *$\Pi_S$ and $\Pi_T$ preserve and reflect labels.*
- *A bijection $\theta : x_T \circledast x_S \cong y_T \circledast y_S$ is in $\cong_{T \circledast S}$ if both $\Pi_T \theta : x_T \cong_T y_T$ and $\Pi_S \theta : x_S \cong_S y_S$.*

*Furthermore, for every $e \in T \circledast S$, at least one of $\Pi_S(e)$ and $\Pi_T(e)$ is defined.*

When reasoning about the polarity of events in $T \circledast S$, a subtlety arises because $B$-moves are not assigned the same polarity in $\mathcal{S}$ and $\mathcal{T}$. This is not surprising: polarity is there precisely to allow strategies to communicate by sending $(+)$ and receiving $(-)$ values; in this interaction, $\mathcal{S}$ and $\mathcal{T}$ play complementary roles. To reason about the flow of information in the event structure $\mathcal{T} \circledast \mathcal{S}$ it will be important, for each $B$-move $e$ of $T \circledast S$, to know whether it is positive in $\mathcal{S}$ or in $\mathcal{T}$; in other words, whether information is flowing *from $\mathcal{S}$ to $\mathcal{T}$*, or vice-versa.

Accordingly, we define $\mathrm{pol}^{\circledast} : T \circledast S \to \{+^{\mathcal{S}}, +^{\mathcal{T}}, 0^{\mathcal{S}}, 0^{\mathcal{T}}, -\}$, as follows:

$$\mathrm{pol}^{\circledast}(e) = \begin{cases} +^{\mathcal{S}} \text{ (resp. } 0^{\mathcal{S}}) & \text{if } \Pi_S(e) \text{ is defined and } \mathrm{pol}(\Pi_S(e)) = + \text{ (resp. 0)} \\ +^{\mathcal{T}} \text{ (resp. } 0^{\mathcal{T}}) & \text{if } \Pi_T(e) \text{ is defined and } \mathrm{pol}(\Pi_T(e)) = + \text{ (resp. 0)}, \\ - & \text{otherwise.} \end{cases}$$

*Probability in the interaction.* Unlike with polarity, $\mathcal{S}$ and $\mathcal{T}$ agree on what measurable space to assign to each $B$-move, since by the conditions on strategies, this is determined by the arena. So for each $e \in T \circledast S$ we can set $\mathcal{M}(e) = \mathcal{M}(\Pi_S(e))$ or $\mathcal{M}(\Pi_T(e))$, unambiguously, and an easy argument shows that this makes $\mathcal{T} \circledast \mathcal{S}$ a well-defined *measurable* event structure with symmetry.

We can turn $\mathcal{T} \circledast \mathcal{S}$ into a quantitative event structure by defining a kernel $k_e^{\circledast} : \mathcal{M}(\mathrm{pa}^{\circledast}(e)) \rightsquigarrow \mathcal{M}(e)$ for every $e \in T \circledast S$ such that $\mathrm{pol}^{\circledast}(e) \neq -$. The key observation is that when $\mathrm{pol}^{\circledast}(e) \in \{+^{\mathcal{S}}, 0^{\mathcal{S}}\}$, the parents of $e$ correspond precisely to the parents of $\Pi_S(e)$ in $\mathcal{S}$. Since $\Pi_S$ preserves the measurable space associated to an event, we may then take $k_e^{\circledast} = k_{\Pi_S(e)}$.

**Hiding.** Hiding is the process of deleting the $B$-moves from $T \circledast S$, yielding a strategy from $\mathcal{A}$ to $\mathcal{C}$. The $B$-moves are exactly those on which both projections are defined, so the new set of events is obtained as follows:

$$T \odot S = \{e \in T \circledast S \mid \Pi_S(e) \text{ and } \Pi_T(e) \text{ are not both defined}\}.$$

This set inherits a preorder $\leq$, conflict relation $\#$, and measurable structure directly from $T \circledast S$. Polarity is lifted from either $S$ or $T$ via the projections. (Note that by removing the $B$-moves we resolved the mismatch.) To define the data flow dependency, we must take care to ensure that the resulting $T \odot S$ is Bayesian. For $e, e' \in T \odot S$, we say $e \dashrightarrow e'$ if one of the following holds:

(1) There exist $n \geq 0$ and $e_1, \ldots, e_n \in T \circledast S$, all $B$-moves, such that $e \dashrightarrow e_1 \dashrightarrow \cdots \dashrightarrow e_n \dashrightarrow e'$ (in $T \circledast S$).
(2) There exist a non-uniform $\mathbf{d} \in T \circledast S$, $n \geq 0$ and $e_1, \ldots, e_n \in T \circledast S$, all $B$-moves, such that such that $e \dashrightarrow e_1 \dashrightarrow \cdots \dashrightarrow e_n \dashrightarrow \mathbf{d}$ and $\mathbf{d} \leq e'$.

From a configuration $x \in \mathscr{C}(T \odot S)$ we can recover the hidden moves to get an **interaction witness** $\overline{x} = \{e \in T \circledast S \mid e \leq e' \in x\}$, a configuration of $\mathscr{C}(T \circledast S)$. For $x, y \in \mathscr{C}(T \odot S)$, a bijection $\theta : x \cong y$ is in $\cong_{T \odot S}$ if there is $\overline{\theta} : \overline{x} \cong_{T \circledast S} \overline{y}$ which restricts to $\theta$. This gives a measurable event structure with symmetry $\mathcal{T} \odot \mathcal{S}$.

To make $\mathcal{T} \odot \mathcal{S}$ a Bayesian event structure, we must define for every $e \in T \odot S$ a kernel $k_e$, which we denote $k_e^{\odot}$ to emphasise the difference with the kernel $k_e^{\circledast}$ defined above. Indeed the parents $\mathrm{pa}^{\circledast}(e)$ of $e$ in $T \circledast S$ may no longer exist in $T \odot S$, where $e$ has a different set of parents $\mathrm{pa}^{\odot}(e)$.

We therefore consider the subset of hidden ancestors of **e** which ought to affect the kernel $k_e^{\odot}$:

**Definition 15.** *For strategies $\mathcal{S} : \mathcal{A} \nrightarrow \mathcal{B}$ and $\mathcal{T} : \mathcal{B} \nrightarrow \mathcal{C}$, and $e \in T \odot S$, an* ***essential hidden ancestor*** *of $e$ is a $B$-move $d \in T \circledast S$, such that $d \leq e$ and one of the following holds:*

*(1) There are $e_1 \in \mathrm{pa}^{\odot}(e), e_2 \in \mathrm{pa}^{\circledast}(e)$ such that $e_1 \dashrightarrow \cdots \dashrightarrow d \dashrightarrow \cdots \dashrightarrow e_2$.*
*(2) There are $e_0 \in \mathrm{pa}^{\odot}(e)$, $B$-moves $d'$ and $e_1, \ldots, e_n$, with $d'$ non-uniform, such that $e_0 \dashrightarrow e_1 \dashrightarrow \cdots \dashrightarrow e_j \dashrightarrow d \dashrightarrow e_{j+1} \dashrightarrow \cdots \dashrightarrow e_n \dashrightarrow d'$.*

Since $\mathcal{T} \odot \mathcal{S}$ is innocent, $e$ has a sequential history, and thus the set of essential hidden ancestors of $e$ forms a finite, total preorder, for which there exists a linear enumeration $d_1 \leq \cdots \leq d_n$. We then define $k_e^{\odot} : \mathcal{M}(\mathrm{pa}(e)) \rightsquigarrow \mathcal{M}(e)$ as follows:

$$k_e^{\odot}(\underline{\mathrm{pa}}^{\odot}(e), U) = \int_{\underline{d}_1} k(\underline{\mathrm{pa}}^{\circledast}(d_1), \mathrm{d}\underline{d}_1) \cdots \int_{\underline{d}_n} k(\underline{\mathrm{pa}}^{\circledast}(d_n), \mathrm{d}\underline{d}_n)\ [k_e^{\circledast}(\underline{\mathrm{pa}}^{\circledast}(e), U)]$$

where we abuse notation: using that for every $i \leq n$, $\mathrm{pa}^{\circledast}(d_i) \subseteq \mathrm{pa}^{\odot}(e) \cup \{d_j \mid j < i\}$, we may write $\underline{\mathrm{pa}}^{\circledast}(d_i)$ for the only element of $\mathcal{M}(\mathrm{pa}^{\circledast}(d_i))$ compatible with $\underline{\mathrm{pa}}^{\odot}(e)$ and $\underline{d}_1, \ldots, \underline{d}_{i-1}$. The particular choice of linear enumeration does not matter by Fubini's theorem for s-finite kernels.

**Lemma 2.** *There is a map $\tau \odot \sigma : \mathcal{T} \odot \mathcal{S} \rightharpoonup \mathcal{A}^{\perp} \parallel \mathcal{C}$ making $\mathcal{T} \odot \mathcal{S}$ a Bayesian strategy. We call this the **composition** of $\mathcal{S}$ and $\mathcal{T}$.*

**Copycat.** We have defined morphisms between arenas, and how they compose. We now define identities, called *copycat strategies*. In the semantics of our language, these are used to interpret typing judgements of the form $x : A \vdash x : A$, and the copycat acts by forwarding values received on one side across to the other. To guide the intuition, the copycat strategy for the game $[\![\mathbb{R}]\!] \multimap [\![\mathbb{R}]\!]$ is pictured in Fig. 8. (We will define the $\multimap$ construction later.)

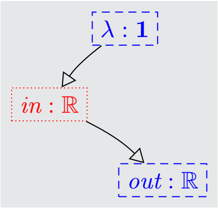

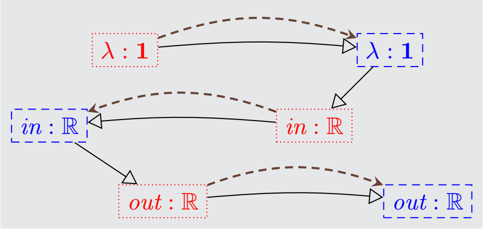

Fig. 8: The arena $[\![\mathbb{R}]\!] \multimap [\![\mathbb{R}]\!]$ (a), and the copycat strategy on it (b).

Formally, the copycat strategy on an arena $\mathcal{A}$ is a Bayesian event structure (with symmetry) $\mathbb{C}\mathbb{C}_{\mathcal{A}}$, together with a (total) map $\mathrm{cc}_{\mathcal{A}} : \mathbb{C}\mathbb{C}_{\mathcal{A}} \to \mathcal{A}^{\perp} \parallel \mathcal{A}$. As should be clear in the example of Fig. 8, the events, polarity, conflict, and measurable structure of $\mathbb{C}\mathbb{C}_A$ are those of $A^{\perp} \parallel A$. The order $\leq$ is the transitive closure of that in $A^{\perp} \parallel A$ enriched with the pairs $\{((a,1),(a,2)) \mid a \in A \text{ and } \mathrm{pol}_A(a) = +\} \cup \{((a,2),(a,1)) \mid \mathrm{pol}_A(a) = -\}$. The same sets of pairs also make up the data dependency relation in $\mathbb{C}\mathbb{C}_A$; recall that there is no data dependency in the event structure $A$. Note that because $\mathbb{C}\mathbb{C}_A$ is just $A^{\perp} \parallel A$ with added constraints, configurations of $\mathbb{C}\mathbb{C}_A$ can be seen as a subset of those of $A^{\perp} \parallel A$, and thus the symmetry $\cong_{\mathbb{C}\mathbb{C}_A}$ is inherited from $\cong_{A^{\perp} \parallel A}$.

To make copycat a Bayesian strategy, we observe that for every positive $e \in \mathbb{C}_A$, $\mathrm{pa}(e)$ contains a single element, the correponding negative move in $A^\perp \parallel A$, which carries the same measurable space. Naturally, we take $k_e : \mathcal{M}(e) \rightsquigarrow \mathcal{M}(e)$ to be the identity kernel.

We have defined objects, morphisms, composition, and identities. They assemble into a category.

**Theorem 1.** *Arenas and Bayesian strategies, with the latter considered up to isomorphism, form a category* **BG**. **BG** *has a subcategory* $\mathbf{BG}^+$ *whose objects are positive, regular arenas and whose morphisms are* ***negative strategies*** *(*i.e. *strategies whose inital moves are negative), up to isomorphism.*

The restriction implies (using receptivity) that for every strategy $\mathcal{A} \nrightarrow \mathcal{B}$ in $\mathbf{BG}^+$, initial moves of $\mathcal{S}$ correspond to $\mathrm{init}(A)$. This reflects the dynamics of a call-by-value language, where arguments are received before anything else. We now set out to define the semantics of our language in $\mathbf{BG}^+$.

## 5 A denotational model

In Sec. 5.1, we describe some abstract constructions in the category, which provide the necessary ingredients for interpreting types and terms in Sec. 5.2.

### 5.1 Categorical structure

The structure required to model a calculus of this kind is fairly standard. The first games model for a call-by-value language was given by Honda and Yoshida [31] (see also [4]). Their construction was re-enacted in the context of concurrent games by Clairambault et al. [20], from whom we draw inspiration. The adaptation is not however automatic as we must account for measurability, probability, data flow, and an interpretation of product types based on coincidences.

**Coproducts.** Given arenas $\mathcal{A}$ and $\mathcal{B}$, their **sum** $\mathcal{A}+\mathcal{B}$ has events those of $A \parallel B$, and inherited polarity, preorder, and measurable structure, but the conflict relation is extended so that $a \# b$ for every $a \in A$ and $b \in B$. The symmetries $\cong_{A+B}, \cong^-_{A+B}$ and $\cong^+_{A+B}$ are restricted from $\cong_{A\parallel B}, \cong^-_{A\parallel B}$ and $\cong^+_{A\parallel B}$.

The arena $\mathcal{A}+\mathcal{B}$ is a coproduct of $\mathcal{A}$ and $\mathcal{B}$ in $\mathbf{BG}^+$. This means that there are injections $\iota_\mathcal{A} : \mathcal{A} \nrightarrow \mathcal{A}+\mathcal{B}$ and $\iota_\mathcal{B} : \mathcal{B} \nrightarrow \mathcal{A}+\mathcal{B}$ behaving as copycat on the appropriate component, and that any two strategies $\sigma : \mathcal{A} \nrightarrow \mathcal{C}$ and $\tau : \mathcal{B} \nrightarrow \mathcal{C}$ induce a unique co-pairing strategy denoted $[\sigma, \tau] : \mathcal{A}+\mathcal{B} \nrightarrow \mathcal{C}$. This construction can be performed for any arity, giving coproducts $\sum_{i\in I} \mathcal{A}_i$.

**Tensor.** Tensor products are more subtle, partly because in this paper we use *coincidence* to deal with pairs, as motivated in Sec. 3.3. For example, given two arenas each having a single initial move, we construct their tensor product by taking their parallel composition and making the two initial moves coincident.

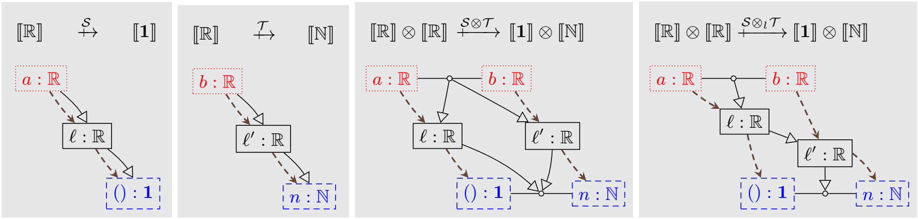

Fig. 9: Example of tensor construction.

More generally, suppose $\mathcal{A}$ and $\mathcal{B}$ are arenas in which all inital events are coincident; we call these **elementary arenas**. Then $A \otimes B$ has all structure inherited from $A \parallel B$, and additionally we set $a \multimap b$ for every $a \in \text{init}(A)$ and $b \in \text{init}(B)$. Since $\mathscr{C}(A \otimes B) \subseteq \mathscr{C}(A \parallel B)$, we can define symmetries on $A \otimes B$ by restricting those in $A \parallel B$.

Now, because arenas in $\mathbf{BG}^+$ are *regular* (Definition 9), it is easy to see that each $\mathcal{A}$ is isomorphic to a sum $\sum_{i \in I} \mathcal{A}_i$ with each $\mathcal{A}_i$ elementary. If $\mathcal{B} \in \mathbf{BG}^+$ is isomorphic to $\sum_{j \in J} \mathcal{B}_j$ with the $\mathcal{B}_j$ elementary, we define $\mathcal{A} \otimes \mathcal{B} = \sum_{i,j} \mathcal{A}_i \otimes \mathcal{B}_j$.

In order to give semantics to pairs of terms, we must define the action of $\otimes$ of strategies. Consider two strategies $\sigma : \mathcal{S} \rightharpoonup \mathcal{A}^\perp \parallel \mathcal{A}'$ and $\tau : \mathcal{T} \rightharpoonup \mathcal{B}^\perp \parallel \mathcal{B}'$. Let $\sigma \parallel \tau : \mathcal{S} \parallel \mathcal{T} \rightharpoonup (\mathcal{A} \parallel \mathcal{B})^\perp \parallel (\mathcal{A}' \parallel \mathcal{B}')$ be defined in the obvious way from $\sigma$ and $\tau$ (note the codomain was rearranged). We observe that $\mathscr{C}((\mathcal{A} \otimes \mathcal{B})^\perp \parallel (\mathcal{A}' \otimes \mathcal{B}')) \subseteq \mathscr{C}((\mathcal{A} \parallel \mathcal{B})^\perp \parallel (\mathcal{A}' \parallel \mathcal{B}'))$ and show:

**Lemma 3.** *Up to symmetry, there is a unique event structure $S \otimes T$ such that $\mathscr{C}(S \otimes T) = \{x \in \mathscr{C}(S \parallel T) \mid (\sigma \parallel \tau)\, x \in \mathscr{C}((\mathcal{A} \otimes \mathcal{B})^\perp \parallel (\mathcal{A}' \otimes \mathcal{B}'))\}$ and such that polarity, labelling, and data flow are lifted from $S \parallel T$ via a projection function $S \otimes T \to S \parallel T$.*

Informally, the strategies synchronise at the start, *i.e.* all initial moves are received at the same time, and they synchronise again when they are both ready to move to the $\mathcal{A}' \otimes \mathcal{B}'$ side for the first time.

The operations $- \otimes \mathcal{B}$ and $\mathcal{A} \otimes -$ on $\mathbf{BG}^+$ define functors. However, as is typically the case for models of call-by-value, the tensor fails to be *bifunctorial*, and thus $\mathbf{BG}^+$ is not monoidal but only *premonoidal* [50]. The unit for $\otimes$ is the arena $\mathbf{1}$ with one (positive) event $() : \mathbf{1}$. There are "copycat-like" associativity, unit and braiding strategies, which we omit.

The failure of bifunctoriality in this setting means that for $\sigma : \mathcal{A} \nrightarrow \mathcal{A}'$ and $\tau : \mathcal{B} \nrightarrow \mathcal{B}'$, the strategy $\mathcal{S} \otimes \mathcal{T}$ is in general distinct from the following two strategies:

$$\mathcal{S} \otimes_l \mathcal{T} = (\mathbb{C}_{\mathcal{A}'} \otimes \mathcal{T}) \odot (\mathcal{S} \otimes \mathbb{C}_{\mathcal{B}}) \qquad \mathcal{S} \otimes_r \mathcal{T} = (\mathcal{S} \otimes \mathbb{C}_{\mathcal{B}'}) \odot (\mathbb{C}_{\mathcal{A}} \otimes \mathcal{T})$$

See Fig. 9 for an example of the $\otimes$ and $\otimes_l$ constructions on simple strategies. Observe that the data flow relation is *not* affected by the choice of tensor: this is related to our discussion of commutativity in Sec. 1.1: a commutative semantics is one that satisfies $\otimes_l = \otimes_r = \otimes$.

We will make use of the left tensor $\otimes_l$ in our denotational semantics, because it reflects a left-to-right evaluation strategy, which is standard. It will also be important that the interpretation of values lies in the **centre** of the premonoidal category, which consists of those strategies $\mathcal{S}$ for which $\mathcal{S} \otimes_l \mathcal{T} = \mathcal{S} \otimes_r \mathcal{T}$ and $\mathcal{T} \otimes_l \mathcal{S} = \mathcal{T} \otimes_r \mathcal{S}$ for every $\mathcal{T}$. Finally we note that $\otimes$ distributes over $+$, in the sense that for every $\mathcal{A}, \mathcal{B}, \mathcal{C}$ the canonical strategy $(\mathcal{A} \otimes \mathcal{B}) + (A \otimes \mathcal{C}) \nrightarrow \mathcal{A} \otimes (\mathcal{B} + \mathcal{C})$ has an inverse $\lambda$.

**Function spaces.** We now investigate the construction of arenas of the form $\mathcal{A} \multimap \mathcal{B}$. This is a *linear* function space construction, allowing at most one call to the argument $\mathcal{A}$; in Sec. 5.1 we will construct an extended arena $!(\mathcal{A} \multimap \mathcal{B})$ permitting arbitrary usage. Given $\mathcal{A}$ and $\mathcal{B}$ we construct $\mathcal{A} \multimap \mathcal{B}$ as follows. (This construction is the same as in other call-by-value game semantics, *e.g.* [31,20].) Recall that we can write $\mathcal{A} = \sum_{i \in I} \mathcal{A}_i$ with each $\mathcal{A}_i$ an elementary arena. Then, $A \multimap B$ has the same set of events as $\mathbf{1} \parallel \sum_{i \in I} (\mathcal{A}_i^{\perp} \parallel \mathcal{B})$, with inherited polarity and measurable structure, but with a preorder enriched with the pairs $\{(\lambda, a) \mid a \in \mathrm{init}(A)\} \cup \{(a_i, (i, b)) \mid a \in \mathrm{init}(A_i), b \in \mathrm{init}(B)\}$, where in this case we call $\lambda$ the unique move of $\mathbf{1}$.

For every strategy $\sigma : \mathcal{A} \otimes \mathcal{B} \nrightarrow \mathcal{C}$ we call $\Lambda(\sigma) : \mathcal{A} \nrightarrow \mathcal{B} \multimap \mathcal{C}$ the strategy which, upon receiving an opening $\mathcal{A}$-move (or coincidence) $\mathbf{a}$, deterministically (and with no data-flow link) plays the move $\lambda$ in $\mathcal{B} \multimap \mathcal{C}$, waits for Opponent to play a $B$-move (or coincidence) $\mathbf{b}$ and continues as $\sigma$ would on input $\mathbf{a} \multimapdot \mathbf{b}$. Additionally there is for every $\mathcal{B}$ and $\mathcal{C}$ an evaluation morphism $\mathrm{ev}_{\mathcal{B},\mathcal{C}} : (\mathcal{B} \multimap \mathcal{C}) \otimes \mathcal{B} \nrightarrow \mathcal{C}$ defined as in [20].

**Lemma 4.** *For a strategy $\sigma : \mathcal{A} \otimes \mathcal{B} \nrightarrow \mathcal{C}$, the strategy $\Lambda(\sigma)$ is central and satisfies* $\mathrm{ev} \odot (\Lambda(\sigma) \otimes \mathrm{cc}) = \sigma$.

**Duplication.** We define, for every arena $\mathcal{A}$, a "reusable" arena $!\mathcal{A}$. Its precise purpose will become clear when we define the semantics of our language. It is helpful to start with the observation that ground type values are readily duplicable, in the sense that there is a strategy $[\![\mathbb{R}]\!] \nrightarrow [\![\mathbb{R}]\!] \otimes [\![\mathbb{R}]\!]$ in **BG**. Therefore ! will have no effect on $[\![\mathbb{R}]\!]$, but only on more sophisticated arenas (*e.g.* $[\![\mathbb{R}]\!] \multimap [\![\mathbb{R}]\!]$) for which no such (well-behaved) map exists. We start by studying *negative* arenas.

**Definition 16.** *Let $\mathcal{A}$ be a* negative *arena. We define $!\mathcal{A}$ to be the measurable event structure $!A = \parallel_{i \in \omega} A$, equipped with the following symmetries:*

- $\cong_{!A}$ *contains those $\theta : \parallel_{i \in \omega} x_i \cong \parallel_{i \in \omega} y_i$ for which there is $\pi : \omega \cong \omega$ and $\theta_i : x_i \cong_{A_i} x_{\pi(i)}$ such that $\theta(a, i) = (\theta_i(a), \pi(i))$ for each $(a, i) \in !A$.*
- $\cong_{!A}^-$ *contains bijections $\theta : x \cong_{!A} y$ such that for each $i \in \omega$, $\theta_i : x_i \cong_A^- y_{\pi(i)}$.*
- $\cong_{!A}^+$ *contains bijections $\theta : x \cong_{!A} y$ s.t. $\pi = \mathrm{id}$ and for each $i$, $\theta_i : x_i \cong_A^+ y_i$.*

It can be shown that $!\mathcal{A}$ is a well-defined negative arena, *i.e.* meets the conditions of Definition 9. Observe that an elementary *positive* arena $\mathcal{B}$ corresponds precisely to a set $\mathbf{e}$ of coincident positive events, all initial for $\multimapdot$, immediately followed by a negative arena which we call $\mathcal{B}_-$. *Followed* here means that $e \leq b$

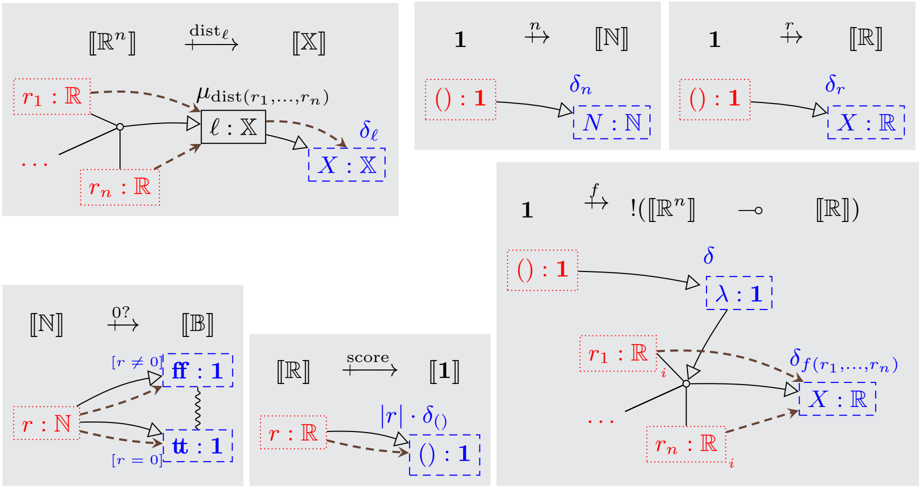

Fig. 10: Constant strategies. (The copy indices $i$ in $f$ indicate that we have $\omega$ symmetric branches.)

for all $e \in \mathbf{e}$ and $b \in B_-$, and we write $\mathcal{B} = \mathbf{e} \cdot \mathcal{B}_-$. We define $!\mathcal{B} = \mathbf{e} \cdot !\mathcal{B}_-$. Finally, recall that an arbitrary positive arena $\mathcal{B}$ can be written as a sum of elementary ones: $\mathcal{B} = \sum_{i \in I} \mathcal{B}_i$. We then define $!\mathcal{B} = \sum_{i \in I} !\mathcal{B}_i$.

For positive $\mathcal{A}$ and $\mathcal{B}$, a *central* strategy $\sigma : \mathcal{A} \nrightarrow \mathcal{B}$ induces a strategy $!\sigma : !\mathcal{A} \nrightarrow !\mathcal{B}$, and this is functorial. The functor $!$ extends to a linear exponential comonad on the category with elementary arenas as objects and central strategies as morphisms (see [20] for the details of a similar construction).

**Recursion.** To interpret fixed points, we consider an ordering relation on strategies. We momentarily break our habit of considering strategies up to isomorphism, as in this instance it becomes technically inconvenient [17].

**Definition 17.** *If $\sigma : \mathcal{S} \rightharpoonup \mathcal{A}$ and $\tau : \mathcal{T} \rightharpoonup \mathcal{A}$ are strategies, we write $\mathcal{S} \sqsubseteq \mathcal{T}$ if $\mathcal{S} \subseteq \mathcal{T}$, the inclusion map is a map of event structures, preserves all structure, including kernels, and for every $s \in S$, $\sigma(s) = \tau(s)$.*

**Lemma 5.** *Every $\omega$-chain $\mathcal{S}_0 \sqsubseteq \mathcal{S}_1 \sqsubseteq \ldots$ has a least upper bound $\bigvee_{i \in \omega} \mathcal{S}_i$, given by the union $\bigcup_{i \in \omega} \mathcal{S}_i$, with all structure obtained by componentwise union.*

There is also a least strategy $\perp$ on every arena, unique up to isomorphism. We are now ready to give the semantics of our language.

### 5.2 Denotational semantics

The interpretation of types is as follows:

$$[\![\mathbf{1}]\!] = \boxed{() : \mathbf{1}} \qquad [\![\mathbb{R}]\!] = \boxed{a : \mathbb{R}} \qquad [\![\mathbb{N}]\!] = \boxed{a : \mathbb{N}}$$

$$\llbracket () \rrbracket^{\Gamma} = \llbracket \Gamma \rrbracket \xrightarrow{\mathrm{w}_\Gamma} \mathbf{1} = \llbracket \mathbf{1} \rrbracket \qquad \llbracket \underline{n} \rrbracket^{\Gamma} = \llbracket \Gamma \rrbracket \xrightarrow{\mathrm{w}_\Gamma} \mathbf{1} \xrightarrow{n} \llbracket \mathbb{N} \rrbracket$$

$$\llbracket \underline{r} \rrbracket^{\Gamma} = \llbracket \Gamma \rrbracket \xrightarrow{\mathrm{w}_\Gamma} \mathbf{1} \xrightarrow{r} \llbracket \mathbb{R} \rrbracket \qquad \llbracket \underline{f} \rrbracket^{\Gamma} = \llbracket \Gamma \rrbracket \xrightarrow{\mathrm{w}_\Gamma} \mathbf{1} \xrightarrow{f} \llbracket \mathbb{R}^n \to \mathbb{R} \rrbracket$$

$$\llbracket x \rrbracket^{\Gamma, x:A} = \llbracket \Gamma \rrbracket \otimes \llbracket A \rrbracket \xrightarrow{\mathrm{w}_\Gamma \otimes \text{œ}_{\llbracket A \rrbracket}} \mathbf{1} \otimes \llbracket A \rrbracket \xrightarrow{\cong} \llbracket A \rrbracket$$

$$\llbracket \lambda x.M \rrbracket^{\Gamma} = \llbracket \Gamma \rrbracket \xrightarrow{h_\Gamma} !\llbracket \Gamma \rrbracket \xrightarrow{!\Lambda\left(\llbracket M \rrbracket^{\Gamma, x:A}\right)} !(\llbracket A \rrbracket \multimap \llbracket B \rrbracket)$$

$$\llbracket M\ N \rrbracket^{\Gamma} = \llbracket \Gamma \rrbracket \xrightarrow{\mathrm{c}_\Gamma} \llbracket \Gamma \rrbracket \otimes \llbracket \Gamma \rrbracket \xrightarrow{\llbracket M \rrbracket^{\Gamma} \otimes_l \llbracket N \rrbracket^{\Gamma}} \llbracket A \to B \rrbracket \otimes \llbracket A \rrbracket$$
$$\xrightarrow{\varepsilon \otimes \text{œ}} (\llbracket A \rrbracket \multimap \llbracket B \rrbracket) \otimes \llbracket A \rrbracket \xrightarrow{\mathrm{ev}} \llbracket B \rrbracket$$

$$\llbracket (M, N) \rrbracket^{\Gamma} = \llbracket \Gamma \rrbracket \xrightarrow{\mathrm{c}_\Gamma} \llbracket \Gamma \rrbracket \otimes \llbracket \Gamma \rrbracket \xrightarrow{\llbracket M \rrbracket^{\Gamma} \otimes_l \llbracket N \rrbracket^{\Gamma}} \llbracket A \times B \rrbracket$$

$$\llbracket \mathbf{match}\ M\ \mathbf{with}\ (x,y) \to N \rrbracket^{\Gamma} = \llbracket \Gamma \rrbracket \xrightarrow{\mathrm{c}_\Gamma} \llbracket \Gamma \rrbracket \otimes \llbracket \Gamma \rrbracket \xrightarrow{\text{œ} \otimes \llbracket M \rrbracket^{\Gamma}} \llbracket \Gamma \rrbracket \otimes \llbracket A \times B \rrbracket \xrightarrow{\llbracket N \rrbracket^{\Gamma, x, y}} \llbracket C \rrbracket$$

$$\llbracket \mathbf{match}\ M\ \mathbf{with}\ [\mathbf{inl}\, x \to N_1 \mid \mathbf{inr}\, x \to N_2] \rrbracket^{\Gamma} = \llbracket \Gamma \rrbracket \xrightarrow{\mathrm{c}_\Gamma} \llbracket \Gamma \rrbracket \otimes \llbracket \Gamma \rrbracket$$
$$\xrightarrow{\text{œ} \otimes \llbracket M \rrbracket^{\Gamma}} \llbracket \Gamma \rrbracket \otimes (\llbracket A_1 \rrbracket + \llbracket A_2 \rrbracket) \xrightarrow{\lambda} \llbracket \Gamma \rrbracket \otimes \llbracket A_1 \rrbracket + \llbracket \Gamma \rrbracket \otimes \llbracket A_2 \rrbracket \xrightarrow{[\llbracket N_1 \rrbracket, \llbracket N_2 \rrbracket]} \llbracket B \rrbracket$$

$$\llbracket M =^{?} 0 \rrbracket^{\Gamma} : \llbracket \Gamma \rrbracket \xrightarrow{\llbracket M \rrbracket^{\Gamma}} \llbracket \mathbb{N} \rrbracket \xrightarrow{0?} \llbracket \mathbb{B} \rrbracket \qquad \llbracket \mathbf{score}\ M \rrbracket^{\Gamma} = \llbracket \Gamma \rrbracket \xrightarrow{\llbracket M \rrbracket^{\Gamma}} \llbracket \mathbb{R} \rrbracket \xrightarrow{\mathbf{score}} \llbracket \mathbf{1} \rrbracket$$

$$\llbracket \mathbf{sample}_\ell\ \mathbf{dist}(M_1, \ldots, M_n) \rrbracket = \llbracket \Gamma \rrbracket \xrightarrow{\mathrm{c}_\Gamma} \llbracket \Gamma \rrbracket \otimes \ldots \otimes \llbracket \Gamma \rrbracket \xrightarrow{\llbracket M_1 \rrbracket^{\Gamma} \otimes_l \ldots \otimes_l \llbracket M_n \rrbracket^{\Gamma}} \llbracket \mathbb{R}^n \rrbracket \xrightarrow{\mathrm{dist}_\ell} \llbracket \mathbb{X} \rrbracket$$

$$\llbracket \mu x.M \rrbracket^{\Gamma} = \bigvee_{i \in \omega} \llbracket M \rrbracket_i^{\Gamma, x}(\bot),$$
$$\text{where } \llbracket M \rrbracket_0^{\Gamma, x}(\bot) = \bot \text{ and } \llbracket M \rrbracket_{n+1}^{\Gamma, x}(\bot) = \llbracket M \rrbracket^{\Gamma, x} \odot (\text{œ}_\Gamma \otimes \llbracket M \rrbracket_n^{\Gamma, x}(\bot)) \odot \mathrm{c}_\Gamma$$

Fig. 11: Interpretation of terms as strategies.

$$\llbracket A + B \rrbracket = \llbracket A \rrbracket + \llbracket B \rrbracket \quad \llbracket A \times B \rrbracket = \llbracket A \rrbracket \otimes \llbracket B \rrbracket \quad \llbracket A \to B \rrbracket = !(\llbracket A \rrbracket \multimap \llbracket B \rrbracket)$$

This interpretation extends to contexts via $\llbracket \cdot \rrbracket = \mathbf{1}$ and $\llbracket x_1 : A_1, \ldots, x_n : A_n \rrbracket = \llbracket A_1 \rrbracket \otimes \ldots \otimes \llbracket A_n \rrbracket$. (In Fig. 7 we used $\llbracket \Gamma \vdash A \rrbracket$ to refer to the arena $\llbracket \Gamma \rrbracket^{\perp} \parallel \llbracket A \rrbracket$.)

A term $\Gamma \vdash M : A$ is interpreted as a strategy $\llbracket M \rrbracket^{\Gamma} : \llbracket \Gamma \rrbracket \to \llbracket A \rrbracket$, defined inductively. For every type $A$, the arena $\llbracket A \rrbracket$ is both a !-coalgebra and a commutative comonoid, so there are strategies $\mathrm{w}_A : \llbracket A \rrbracket \nrightarrow \mathbf{1}$, $\mathrm{c}_A : \llbracket A \rrbracket \nrightarrow \llbracket A \rrbracket \otimes \llbracket A \rrbracket$, and $h_A : \llbracket A \rrbracket \nrightarrow !\llbracket A \rrbracket$. Using that the comonad ! is monoidal, this structure extends to contexts; we write $\mathrm{c}_\Gamma, \mathrm{w}_\Gamma$ and $h_\Gamma$ for the induced maps. The interpretation of constants is shown in Fig. 10, and the rest of the semantics is given in Fig. 11.

**Lemma 6.** *For a value $\Gamma \vdash V : A$, the strategy $\llbracket V \rrbracket^{\Gamma}$ is central.*

The semantics is sound for the usual call-by-value equations.

**Proposition 1.** *For arbitrary terms $M, P, N_1, N_2$ and values $V, W$,*

$$\llbracket (\lambda x.M)\, V \rrbracket^{\Gamma} = \llbracket M[V/x] \rrbracket^{\Gamma}$$
$$\llbracket \mathbf{match}\ (V, W)\ \mathbf{with}\ (x, y) \to P \rrbracket^{\Gamma} = \llbracket P[V/x][W/y] \rrbracket^{\Gamma}$$
$$\llbracket \mathbf{match}\ \mathbf{inl}\, V\ \mathbf{with}\ [\mathbf{inl}\, x \to N_1 \mid \mathbf{inr}\, x \to N_2] \rrbracket^{\Gamma} = \llbracket N_1[V/x] \rrbracket^{\Gamma}.$$

The equations are directly verified. Standard reasoning principles apply given the categorical structure we have outlined above. (It is well known that premonoidal categories provide models for call-by-value [50], and our interpretation is a version of Girard's translation of call-by-value into linear logic [29].)

## 6 Conclusion and perspectives

We have defined, for every term $\Gamma \vdash M : A$, a strategy $[\![M]\!]^\Gamma$. This gives a model for probabilistic programming which provides an explicit representation of data flow. In particular, if $\vdash M : \mathbf{1}$, and $M$ has no subterm of type $B + C$, then the Bayesian strategy $[\![M]\!]$ is a Bayesian network equipped with a total ordering of its nodes: the control flow relation $\leq$. Our proposed compositional semantics additionally supports sum types, higher types, and open terms.

This paper does not contain an adequacy result, largely for lack of space: the 'Monte Carlo' operational semantics of probabilistic programs is difficult to define in full rigour. In further work I hope to address this and carry out the integration of causal models into the framework of [53]. The objective remains to obtain proofs of correctness for existing and new inference algorithms.

*Related work on denotational semantics.* Our representation of data flow based on coincidences and a relation $\dashrightarrow$ is novel, but the underlying machinery relies on existing work in concurrent game semantics, in particular the framework of games with symmetry developed by Castellan et al. [17]. This was applied to a language with *discrete* probability in [15], and to a *call-by-name* and *affine* language with continuous probability in [49]. This paper is the first instance of a concurrent games model for a higher-order language with recursion and continuous probability, and the first to track internal sampling and data flow.

There are other interactive models for statistical languages, e.g. by Ong and Vákár [47] and Dal Lago et al. [38]. Their objectives are different: they do not address data flow (*i.e.* their semantics only represents the control flow), and do not record internal samples.

Prior to the development of probabilistic concurrent games, probabilistic notions of event structures were considered by several authors (see [58,1,59]). The literature on probabilistic Petri nets important related work, as Petri nets can sometimes provide finite representations for infinite event structures. *Markov nets* [7,2] satisfy conditional independence conditions based on the causal structure of Petri nets. More recently Bruni et al. [12,13] relate a form of Petri nets to Bayesian networks and inference, though their probability spaces are discrete.

*Related work on graphical representations.* Our event structures are reminiscent of Jeffrey's graphical language for premonoidal categories [35], which combines string diagrams [36] with a control flow relation. Note that in event structures the conflict relation provides a model for sum types, which is difficult to obtain in Jeffrey's setting. The problem of representing sum types arises also in probabilistic modelling, because Bayesian networks do not support them: [45] propose an extended graphical language, which could serve to interpret first-order probabilistic programs with conditionals. Another approach is by [42], whose Bayesian networks have edges labelled by predicates describing the branching condition. Finally, the theory of Bayesian networks has also been investigated extensively by Jacobs [34] with a categorical viewpoint. It will be important to understand the formal connections between our work and the above.
